# Exploring infodemiology: unraveling the intricate relationships among stress, headaches, migraines, and suicide through Google Trends analysis

**DOI:** 10.3389/fdata.2024.1365417

**Published:** 2025-01-07

**Authors:** Rapuru Rushendran, Vellapandian Chitra

**Affiliations:** Department of Pharmacology, SRM College of Pharmacy, SRM Institute of Science and Technology, Chengalpattu, Tamil Nadu, India

**Keywords:** Google Trends, public behavior, internet search, public health, public survey

## Abstract

**Introduction:**

Google Trends has emerged as a vital resource for understanding public information-seeking behavior. This study investigates the interconnected search trends of stress, headaches, migraines, and suicide, highlighting their relevance to public health and mental well-being. By employing infodemiology, the study explores temporal and geographical patterns in search behavior and examines the impact of global events like the COVID-19 pandemic.

**Methods:**

Data mining was conducted using Google Trends for the search terms “stress,” “headache,” “migraine,” and “suicide.” Relative Search Volume (RSV) data from October 2013 to October 2023 was collected and adjusted for time and location. Statistical analyses, including Pearson correlation tests, linear regression, and seasonal Mann-Kendall tests, were applied to identify correlations, trends, and seasonal variations. Geographical differences were also analyzed to understand regional disparities.

**Results:**

Significant correlations were observed among the search terms, with “migraine” and “suicide” showing the strongest association. Seasonal variations revealed a peak in search volumes during winter months. Geographical analysis highlighted consistently high RSV in the Philippines for all terms. During the COVID-19 pandemic, searches for stress, headaches, and migraines showed notable increases, reflecting heightened public interest in mental health-related topics during this period.

**Discussion:**

The study underscores the interconnected nature of stress, headaches, migraines, and suicide in public search behavior. Seasonal patterns and regional variations emphasize the need for targeted interventions. The observed surge in search volume during the COVID-19 pandemic highlights the profound impact of global crises on mental health and the importance of timely public health responses.

**Conclusion:**

Google Trends provides valuable insights into the public's interest in health-related topics, demonstrating the intricate relationship between stress, headaches, migraines, and suicide. The findings highlight the need for increased mental health awareness and interventions, particularly during times of heightened stress. Further research is essential to develop strategies that mitigate the impact of these stressors on public health.

## 1 Background

In today's digital age, the internet is a valuable source of information and expertise, answering our inquiries instantly. Typing keywords into a search engine like Google brings up a massive library of info for many people. This powerful tool can provide insights, direction, and reassurance by connecting people to a world of information. This examines public search utilizing Google and how people use the internet to learn about stress, headaches, migraines, and suicide. The internet is full of knowledge, but disinformation, stigma, and sensitive topics provide distinct issues. Human health and wellbeing have complex links that often go unnoticed. The intricate relationship between stress, headaches, migraines, and suicide is one example. This complex interaction shows how our physical and mental health may affect each other in fundamental and sometimes devastating ways. Stress is a physiological and psychological response to challenging or adverse situations. It can trigger various physical and mental health issues, including headaches and migraines (Burrowes et al., 2022; Minen et al., 2016; Radat, 2013; Schneiderman et al., 2005). Stress can be caused by professional, personal, or financial issues. Stress can cause tension-type headaches. Stress can produce or worsen stress-induced headaches, which are dull, continuous pain (Chowdhury, 2012; McGeary et al., 2022; Panerai, 2012). Reducing stress via relaxation and lifestyle modifications helps reduce headaches. Migraines are neurological conditions that cause severe headaches, nausea, vomiting, and light and sound sensitivity. Stress may cause migraines in some people, but the exact association is unknown (Aguilar-Shea et al., 2022; Ha and Gonzalez, 2019; Peters, 2019). Migraines can severely impact life quality. Suicide from mental and emotional stress is devastating. Stress, headaches, and migraines may lead to suicide, although not always. Migraines can cause sadness and suicidal thoughts due to their daily impact. Stress and mental health issues are linked to headaches, migraines, and suicide, thus they must be addressed to manage these conditions (Brådvik, 2018; Giakas et al., 2023). People with these concerns should get help from mental health professionals and headache and migraine specialists. Early stress management can lower the likelihood of serious consequences, including suicidal thoughts and behaviors (Colizzi et al., 2020). Stress plagues modern society. All genders, ages, and socioeconomic classes are vulnerable. The American Psychological Association reports that around 80% of Americans have experienced at least one indicator of stress, showing that stress levels in the US have remained high (Canady, 2021). Migraines were the biggest cause of impairment in women under 50, according to the 2019 Global Burden of Disease report (Stovner et al., 2022). Only 12% of the world's population has migraines, but that's still a lot. Women have more migraines than men. Migraines affect 18% of women and 6% of men, however women are more likely (Peterlin et al., 2011). For migraine and headache prevention and treatment, stress management and other lifestyle factors are important. Stress management, healthy lifestyle choices, and prompt medical care can reduce the detrimental impacts of these disorders. Infodemiology, a new field, blends health informatics, clinical research, and patient views. It examines how focused internet searches for user-generated health content can benefit public health. Web searches can reveal people's opinions, habits, interests, and savviness (Effenberger et al., 2021; Eysenbach, 2020). The first study linked online searches to search phrases. Over the past decade, infodemiological research has increasingly used Twitter and Google. Numerous researchers have used infodemiology to study many health issues (Mavragani, 2020). Additionally, these recordings are becoming useful for understanding social behavior. Infodemiology's capacity to collect quantitative and qualitative data in real time automatically and affordably is its strength. Google Trends helps track and understand search query and topic popularity. It helps individuals, businesses, researchers, and journalists make educated decisions, develop relevant content, and keep up with digital trends (Gupta et al., 2022; Mackey et al., 2022; Zeraatkar and Ahmadi, 2018). Stress, headaches, migraine, and suicide are extremely common and interconnected robustly. Google Trends will be used to analyse the global association between stress, headache, migraine, suicide and their temporal pattern over 10 years (2013–2023). This study provides unique insights into health-related search behaviors by integrating physical health terms like “headache” and “migraine” with mental health indicators such as “stress” and “suicide,” offering a holistic perspective on the interplay between physical and mental health. Unlike prior studies that often focus narrowly on mental health-related terms, this research examines regional variations, highlighting how cultural, social, and economic factors influence search trends across different countries. Additionally, it explores temporal spikes and seasonal patterns in search behavior, shedding light on fluctuations linked to societal and global events, such as the COVID-19 pandemic. By employing multivariate regression, the study provides a statistically robust analysis of the relationships between terms while accounting for potential confounders, extending beyond descriptive analyses commonly found in infodemiology. These elements differentiate this work and contribute to a deeper understanding of public health trends.

## 2 Methods

### 2.1 Search strategy

We utilized the Google Trends dataset to conduct data mining on a sample of searches like “stress,” “headache,” “migraine,” and “suicide.” Google Trends is a free tool that provides access to multi-timeline data, geographical maps, and top-related queries on a global or national scale, categorized by various search criteria which gives insights on search trends in the unit of Relative Search Volume (RSV). Data was collected monthly from October 2013 to October 2023 and subsequently exported into Microsoft Excel. The numerical data obtained was then graphed for visualization. To facilitate the comparison of these terms and their relationships, we adjusted the Google Trends data proportionally based on both the time and location of each query. This adjustment was performed by dividing the value for each term by the total number of searches within the relevant geographical and time range. This ensured that regions with the highest raw search volume were not necessarily ranked the highest in terms of search interest. The results of these calculations were then transformed into a scaled format, ranging from 0 to 100, based on the relative proportions of these terms to all searches across various topics. This scaling approach allowed for the fair comparison of different regions, even if they had the same raw number of searches for a particular term, as it accounted for variations in search volume (Pullan and Dey, 2021; Swerts et al., 2022). This study utilized Google Trends data, which provides RSVs to assess the proportion of searches for specific terms relative to all search activity in a given region and time frame. RSVs are normalized values ranging from 0 to 100 and do not reflect absolute search counts. While RSVs allow for comparisons of search interest across regions and over time, they do not provide information on the exact number of searches or the specific motivations behind these searches.

### 2.2 Statistical analysis

We conducted an analysis of data extracted from Google Trends, wherein we compared the search volume data provided by the database. Following an initial descriptive analysis involving dispersion diagrams, we employed the Pearson test to assess the correlation between search volumes for stress, headache, migraine, and suicide. To minimize potential confounding variables, we used the adjusted R-squared statistic to evaluate how closely the data aligned with the regression line. Statistical computations were carried out using the GraphPad Prism 8.4.3 software, and we considered p < 0.05 as the threshold for statistical significance. In cases where a strong Pearson correlation was identified, a linear regression model was employed to estimate the search volume for the terms. We calculated a 95% confidence interval (CI) to gauge the predictive capacity of rhinitis search volume in determining the search volume for the specified terms. Google Trends does not give absolute search volume data, which is something we are aware of when it comes to determining the total number of searches. Instead, RSVs show how many times a phrase appeared in Google searches compared to all searches within a certain time period. It is not intended to record exact volumes, but rather patterns and trends in search behavior. To learn more about how people feel about stress, migraines, headaches, and suicide, we looked at how RSVs changed relative to each other over time. The RSV was provided as mean, standard deviation in the country study. The seasonal Mann-Kendall test (alpha-0.05) was used to determine the presence of a significant trend in the study period by XLSTAT statistical software for excel. We used linear regression to calculate the slope as changes in RSV per year and the percentage of mean of RSV per year of significant trends. We utilized R^2^ values to evaluate the strength of correlations between search terms such as stress, headache, migraine, and suicide. While R^2^ > 0.60 was considered strong, R^2^ values between 0.30 and 0.60 were interpreted as moderate, and R^2^ < 0.30 as weak, based on established conventions in behavioral and social sciences. A moderate correlation e.g., stress and suicide with R^2^ = 0.40 suggests a meaningful relationship, particularly when contextualized with public health implications. Practical relevance was determined by considering both statistical significance (*p* < 0.05) and the potential impact of the observed trends on public health. For instance, even moderate correlations were considered practically relevant if they aligned with known health phenomena, such as the association between stress and suicide. These thresholds were established to balance statistical rigor with the exploratory nature of using Google Trends data for public health insights. The multivariate analysis was conducted to examine the relationships between stress, headache, and migraine RSVs (independent variables) and suicide RSV (dependent variable) using Ordinary Least Squares regression. Model performance was assessed using R^2^, adjusted R^2^, F-statistic, and the Durbin-Watson statistic to evaluate fit, significance, and autocorrelation (Le et al., 2023a,b; Rahul et al., 2023; Swerts et al., 2022).

## 3 Results

### 3.1 Exploring the relationship between stress, headaches, migraines, and suicide

**Figure 1:**
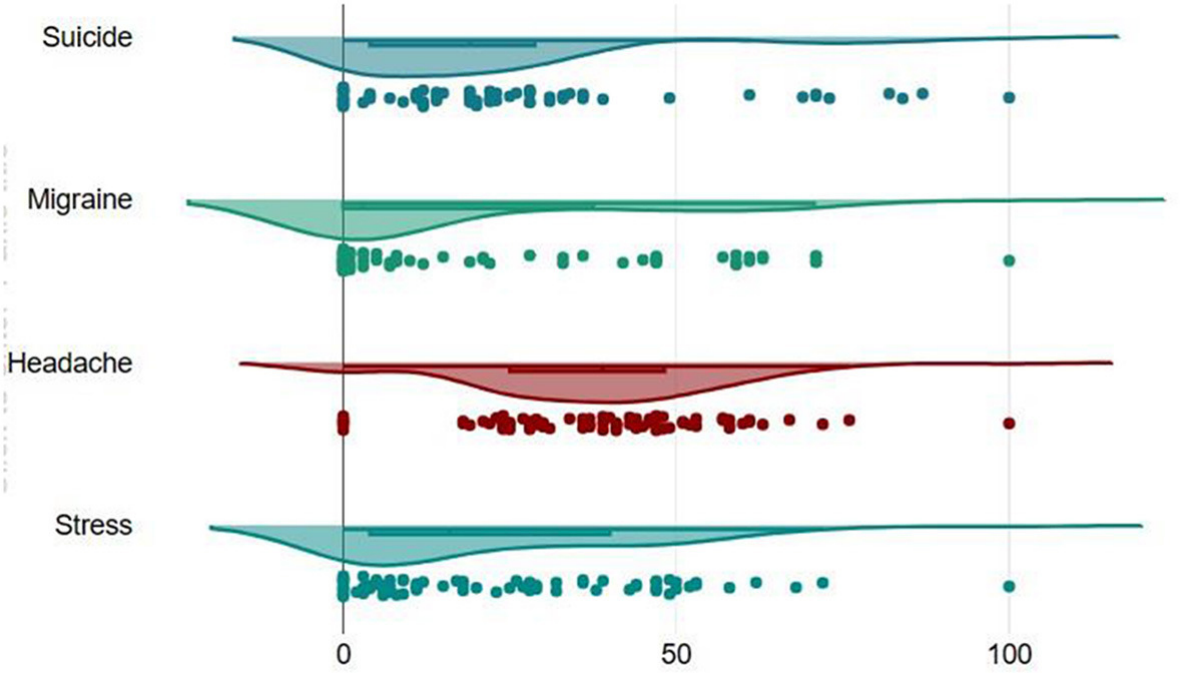
Trends in search terms, analyzed on a country-by-country 2013–2023.

**Figure 2:**
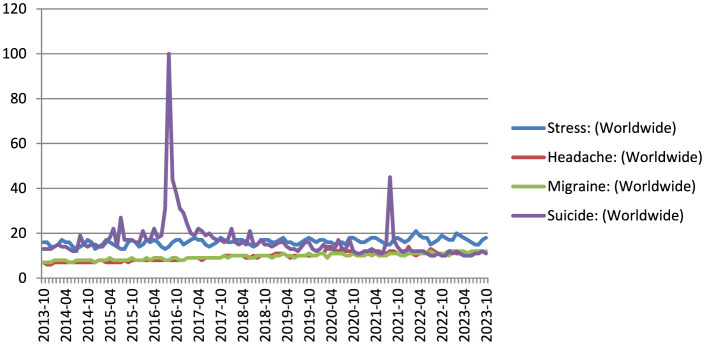
The global relative search volume for stress, headache, migraine, and suicide over the last decade (2013–23).

**Figure 3:**
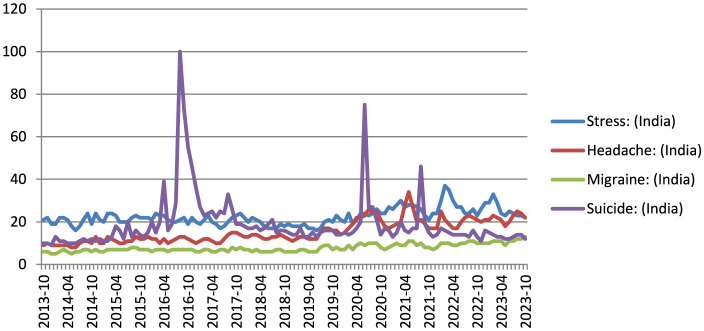
The relative search volume for stress, headache, migraine, and suicide in India over the past decade (2013–23).

We evaluated the association between the RSVs of the search terms stress, headache, migraine, and suicide during the study period. We found strong correlation between the search terms and suggested that the terms trends were correlated. Figure 1 represents the dispersion diagram of all the key terms used in the study. The Pearson correlation coefficient for stress with headache, migraine, suicide was 0.09, 0.77, 0.63 with an adjusted R-squared of 0.008, 0.60, 0.40 (*p*-value:0.43^ns^, < 0.0001^****^, < 0.0001^****^); headache with stress, migraine, suicide was 0.09, 0.11, 0.28 with an adjusted R-squared of 0.008, 0.013, 0.08 (*p*-value:0.43^ns^, 0.31^ns^, 0.01^*^); migraine with stress, headache, suicide was 0.77, 0.11, 0.70 with an adjusted R-squared of 0.60, 0.013, 0.49 (*p*-value: < 0.0001^****^, 0.31^ns^, < 0.0001^****^); suicide with stress, headache, migraine was 0.63, 0.28, 0.70 with an adjusted R-squared of 0.40, 0.08, 0.49 (*p*-value: < 0.0001^****^, 0.0118^*^, < 0.0001^****^). The value of p < 0.05 in the linear regression test indicates that the linear regression model applied has a good predictor of the variables such as stress, headache and suicide search volume. The correlation between the stress, headache, migraine, and suicide. Figures 2, 3 show the survey volume of the terms on the Google Trends platform. From the period of October 2013 to October 2023, a seasonal variation in the search for these terms was correlated every year. Additionally, from month to month, there appears to be a strong correlation between these four search queries. Most notably, however, is the fact that throughout this time period, the searches for suicide rapidly increased at a fraction of the rate in 2016–17 and during Covid-19 period (2019–21) and it was strongly correlated in the past 10 years. It suggests an increase in people experiencing headache and migraine like conditions mainly due to stress worldwide and India. Based on this report it reveals that most of the suicidal thoughts behavior was observed in Indian population compare to worldwide.

### 3.2 Analyzing time series data related to stress, headache, migraine, and suicidal incidents

The study found that the RSVs of terms showed persistent seasonal variations from October 2013–2023. During the winter season there was surge in searches, but in Nations like Bolivia, Costa Rica, Dominican Republic, Ecuador, Guatemala, Iraq, Kazakhstan, Kuwait, and Slovakia the country wise time series analysis found no seasonal variation for the term stress, headache, and suicide. Philippines, South Africa, United States, United Kingdoms, Australia, India, etc. shown seasonal variation for all the terms worldwide listed in Table 1 and the time-series data encompassing the years 2013 to 2023 was shown in Table 2. Among the selected countries Philippines observed most RSV in all the searched terms such as stress-100, headache-63, migraine-100, and suicide-71.

**Table 1:** Search term trends by country spanning from 2013 to 2023.

**Country**	**Stress**	**Headache**	**Migraine**	**Suicide**
Algeria	18	19	21	00
Argentina	05	30	00	14
Australia	53	47	59	87
Austria	25	41	00	20
Bangladesh	15	00	21	28
Belgium	44	37	61	33
Bolivia	00	43	00	00
Brazil	08	57	01	07
Bulgaria	00	21	00	00
Canada	50	51	63	84
Chile	06	45	00	28
Colombia	04	41	00	12
Costa Rica	00	46	00	00
Croatia	00	29	00	22
Czechia	09	29	00	19
Denmark	68	34	00	25
Dominican Republic	00	36	00	00
Ecuador	00	39	00	00
Egypt	12	48	08	14
Finland	11	36	00	28
France	36	24	59	49
Germany	29	46	03	19
Ghana	53	00	71	00
Greece	11	24	00	19
Guatemala	00	48	00	00
Hong Kong	26	40	15	28
Hungary	07	31	00	19
India	27	24	33	33
Indonesia	18	51	03	15
Iran	06	72	00	09
Iraq	00	25	00	00
Ireland	49	58	63	71
Israel	09	29	08	14
Italy	28	36	01	22
Jamaica	72	00	00	00
Japan	02	100	01	03
Jordan	00	47	00	00
Kazakhstan	00	25	00	00
Kenya	58	67	45	36
Kuwait	00	53	00	00
Lebanon	38	00	42	00
Malaysia	47	47	36	39
Mexico	04	44	01	22
Morocco	20	18	22	20
Nepal	39	00	57	00
Netherlands	50	53	71	23
New Zealand	44	49	61	82
Nigeria	52	41	47	28
Norway	43	41	10	36
Pakistan	32	28	47	31
Peru	06	39	00	11
Philippines	100	63	100	71
Poland	05	37	00	11
Portugal	23	24	00	23
Romania	09	25	00	22
Russia	03	23	01	04
Saudi Arabia	11	76	12	12
Serbia	00	28	00	19
Singapore	49	52	59	73
Slovakia	00	30	00	00
South Africa	62	61	47	61
South Korea	17	22	07	11
Spain	06	27	03	11
Sri Lanka	29	0	33	34
Sweden	47	39	07	31
Switzerland	36	37	19	26
Taiwan	07	43	03	04
Thailand	07	58	05	12
Tunisia	28	58	28	00
Turkey	03	00	01	14
Ukraine	03	18	00	04
United Arab Emirates	32	44	47	36
United Kingdom	47	57	71	69
United States	47	61	71	100
Venezuela	00	28	00	12
Vietnam	18	60	05	04

**Table 2:** Analyzing the time series data for the keywords stress, headache, migraine, and suicide from 2013 to 2023.

**Search term**	**Location**	**Mean**	**SD**	**Tau**	**Sens slope**	***p*-value**
Stress	Worldwide	39.31405	3.690151	0.867	1.333	0.001
Stress	India	60.80165289	10.54642106	0.378	0.203	0.152
Headache	Worldwide	72.06612	12.78784	1	0.248	< 0.0001
Headache	India	46.12396694	15.63999694	0.911	0.238	0.000
Migraine	Worldwide	9.727272727	1.367387253	0.956	2.195	0.000
Migraine	India	63.33057851	14.18472678	0.778	0.209	0.002
Suicide	Worldwide	16.68595041	9.747566672	−0.600	−0.619	0.020
Suicide	India	18.76033058	12.6931903	−0.111	−0.123	0.721

### 3.3 Examining the trends in search queries for stress, headache, migraine, and suicide during the COVID-19 pandemic

We evaluated RSVs of the search terms pre, during, and post Covid-19. It was observed that there were significant changes in the RSVs of stress (*p*-value: 0.0006), headache (*p*-value: 0.0007), migraine (*p*-value: 0.002), and suicide (*p*-value: 0.15) during Covid-19 represented in the Table 3 and dominant search inquiries associated with the particular search term over the last 10 years was listed in Table 4. The study further observed an increase in RSV during Covid-19 period RSV 80-100. After that there was a peak observed in search volume during the other waves of Covid-19. Interestingly, the study found that search interest was high in some countries like Kenya, Philippines, Jamaica, Trinidad and Tobago, United States, and Netherlands.

**Table 3:** Assessing the mean relative search volume for the search terms “stress,” “headache,” and “migraine” before, during, and after the COVID-19 pandemic.

**Search term**	**Pre Covid-19 2013–2019**	**During Covid-19 2020–2021**	**Post Covid-19**	***P* value 2022–2023**
Stress	87.53 ± 7.70	86.16 ± 6.38	80.67 ± 8.24	0.0006^***^
Headache	80.78 ± 11.52	74.07 ± 6.38	75.65 ± 5.54	0.0007^***^
Migraine	79.68 ± 10.28	92.58 ± 4.49	89.15 ± 4.06	0.0020^**^
Suicide	18.69 ± 11.07	21.25 ± 11.79	69.85 ± 7.57	0.1589

^***^Highly significant (*P* ≤ 0.001), ^**^Significant (*P* ≤ 0.01).

**Table 4:** Prominent search queries linked to the specific search term over the past decade (2013–2023).

**Top querries**	**RSV**	**Top countries (RSV)**	
**Stress**			
Stress test	100	United States	100
		Denmark	95
		Canada	93
What is stress	86	Philippines	100
		Ethiopia	79
		Zambia	72
Anxiety	55	Australia	100
		Ireland	100
		United Kingdom	97
Stress symptoms	43	South africa	100
		Zimbabwe	93
		Trinidad and Tobago	72
Stress fracture	36	United States	100
		Australia	93
		Ireland	74
**Headache**			
Headache	100	Jamaica	100
		Trinidad and Tobago	97
		Kenya	90
Headaches	35	Trinidad and Tobago	100
		Jamaica	72
		South Africa	63
Migraine	4	Philippines	100
		Netherlands	75
		United States	71
Nausea	4	South Africa	100
		Kenya	94
		United States	91
Headache nausea	3	South Africa	100
		United States	86
		Trinidad and Tobago	80
**Migraine**			
Migraine headache	100	Zambia	100
		Zimbabwe	93
		Nepal	80
Headache	98	Jamaica	100
		Kenya	96
		Trinidad and Tobago	93
Migraine symptoms	79	Philippines	100
		Nepal	83
		United Kingdom	66
Migraines	44	United States	100
		United Kingdom	73
		Canada	69
Migraine pain	41	Pakistan	100
		Bangladesh	74
		United Kingdom	46
**Suicide**			
Suicide squad	100	Puerto Rico	100
		Philippines	95
		United States	95
The suicide squad	13	Australia	100
		United States	98
		United Kindom	96
Commit suicide	13	Singapore	100
		South Africa	92
		Jamaica	74
How to suicide	11	Philippines	100
		South Africa	96
		Australia	72
Suicide squad 2	9	United States	100
		United Kindom	87
		Australia	87

### 3.4 Regional variations

The analysis of Relative Search Volumes (RSVs) highlights notable regional trends, reflecting the influence of cultural attitudes, healthcare access, and societal pressures on online health-seeking behavior. High RSVs in the Philippines for stress (44%) and suicide (21%) suggest significant public interest, likely driven by cultural stigma and limited access to mental health services. Similarly, India exhibits balanced RSVs for stress (37%), headache (26%), and suicide (27%), pointing to rising concerns about mental health amidst urbanization and healthcare inequities, while a lower RSV for migraines (10%) may indicate underreporting or limited awareness. In the United States, suicide (37%) stands out as a key search term, potentially reflecting active mental health discourse and widespread digital health tools, with moderate RSVs for stress (28%), headache (25%), and migraine (10%) indicating robust health literacy. Moderate RSV regions, such as Nigeria and South Africa, reveal significant interest in stress and headaches (45% and 32% in Nigeria; 39% and 30% in South Africa), underscoring the burden of lifestyle-related challenges and socio-economic pressures. In Kenya, RSVs for stress (40%), headache (38%), and suicide (15%) point to a significant overlap of mental and physical health challenges, with low RSVs for migraine (7%) reflecting limited awareness or diagnostic capabilities. Conversely, low RSV regions, such as Denmark and Germany, show high RSVs for stress (75% and 66%) but low for headache (5%), migraine (1%-2%), and suicide (19%-29%), suggesting effective healthcare systems that reduce online dependency for specific conditions. Similarly, Vietnam and Indonesia exhibit high RSVs for stress (74%-56%) but lower for migraine (2%-5%) and suicide (14%-33%), potentially influenced by cultural factors or limited mental health discourse. Globally, stress emerges as a universal concern, consistently registering the highest RSVs across all regions. However, the low RSVs for migraines highlight a potential lack of differentiation between headaches and migraines or limited understanding of the neurological condition. High-pressure societal norms in nations like Italy, France, Japan, South Korea, and Thailand also drive significant RSVs for stress and suicide, reflecting an urgent need for targeted mental health interventions. These insights emphasize the importance of considering cultural and healthcare influences when interpreting regional RSV disparities and their implications for public health strategies.

### 3.5 Public health recommendations and interventions

Based on the findings of this study, we propose the following targeted public health interventions to address the interconnected issues of stress, headaches, migraines, and suicide, while accounting for regional variations in search trends. In regions like the Philippines, India, and the United States, where high Relative Search Volumes (RSVs) for stress and suicide were observed, culturally tailored awareness campaigns should be launched to reduce stigma surrounding mental health. These campaigns can include public service announcements, social media outreach, and collaborations with local influencers to normalize conversations about mental health and encourage seeking professional support. Leverage digital tools such as mobile health apps, telemedicine platforms, and virtual counseling services in regions with high internet use but limited mental health access, such as the Philippines, South Africa, and India. These tools can provide accessible and cost-effective solutions for individuals hesitant or unable to access in-person mental health services. Develop community-focused stress management programs in countries like Kenya and Nigeria, where significant RSVs for stress and headache indicate the need for interventions at the grassroots level. These programs can include mindfulness workshops, physical activity classes, and peer-support groups aimed at reducing stress and improving mental wellbeing. In countries with high RSVs for stress, such as Vietnam, Indonesia, and Nigeria, schools and workplaces can implement stress-reduction strategies. These initiatives can include training for teachers and managers on early identification of mental health issues, providing on-site counseling, and organizing wellness programs focused on work-life balance and stress management techniques. Strengthen mental health care infrastructure in regions like India, South Africa, and Nigeria by training healthcare providers to identify and address comorbid mental health conditions, such as anxiety and depression, particularly in patients presenting with headaches or migraines. This intervention can include the integration of mental health screenings into primary healthcare settings. In countries with high RSVs for suicide, such as the United States, Japan, and Thailand, comprehensive suicide prevention strategies should be prioritized. These can include setting up accessible crisis helplines, community-based mental health first aid training, and integration of suicide risk assessment into routine medical check-ups. Utilize Google Trends and other digital surveillance tools to monitor spikes in search interest related to stress, migraines, and suicide. Governments and public health agencies can use this real-time data to deploy timely interventions, such as increased availability of crisis counseling during peak periods or in response to external stressors like pandemics or economic downturns. To address the low RSVs for migraines in many regions, public health agencies should develop educational campaigns to differentiate migraines from general headaches, promote early diagnosis, and raise awareness about available treatment options. These campaigns should focus on both urban and rural areas to bridge the awareness gap. These interventions aim to reduce the burden of stress-related conditions and suicide while enhancing public mental health literacy and access to care. By addressing local needs through culturally sensitive and evidence-based strategies, these recommendations can inform policymakers and public health officials in developing effective, targeted solutions.

### 3.6 Multivariate analysis

The multivariate regression analysis assessed the relationships between suicide RSV (dependent variable) and three independent variables: stress RSV, headache RSV, and migraine RSV. The model demonstrated a high explanatory power, with R^2^ = 0.997, indicating that 99.7% of the variance in suicide RSV is explained by the independent variables. The adjusted R^2^ value of 0.997 confirms the robustness of the model after accounting for the number of predictors.

1. **Stress RSV**: a significant negative relationship (β = −0.991, *p* < 0.0001), where a 1% increase in stress RSV is associated with a 0.991% decrease in suicide RSV, holding other variables constant.
2. **Headache RSV**: a significant negative relationship (β = −1.002, *p* < 0.0001), indicating a 1% increase in headache RSV corresponds to a 1.002% decrease in suicide RSV.
3. **Migraine RSV**: a significant negative relationship (β = −0.993, *p* < 0.0001), where a 1% increase in migraine RSV predicts a 0.993% decrease in suicide RSV.

The F-statistic (*p* < 0.0001) confirms that the model as a whole is statistically significant. Additionally, the Durbin-Watson statistic (2.098) suggests no autocorrelation in the residuals, validating the independence of observations.

## 4 Discussion

**Figure 4:**
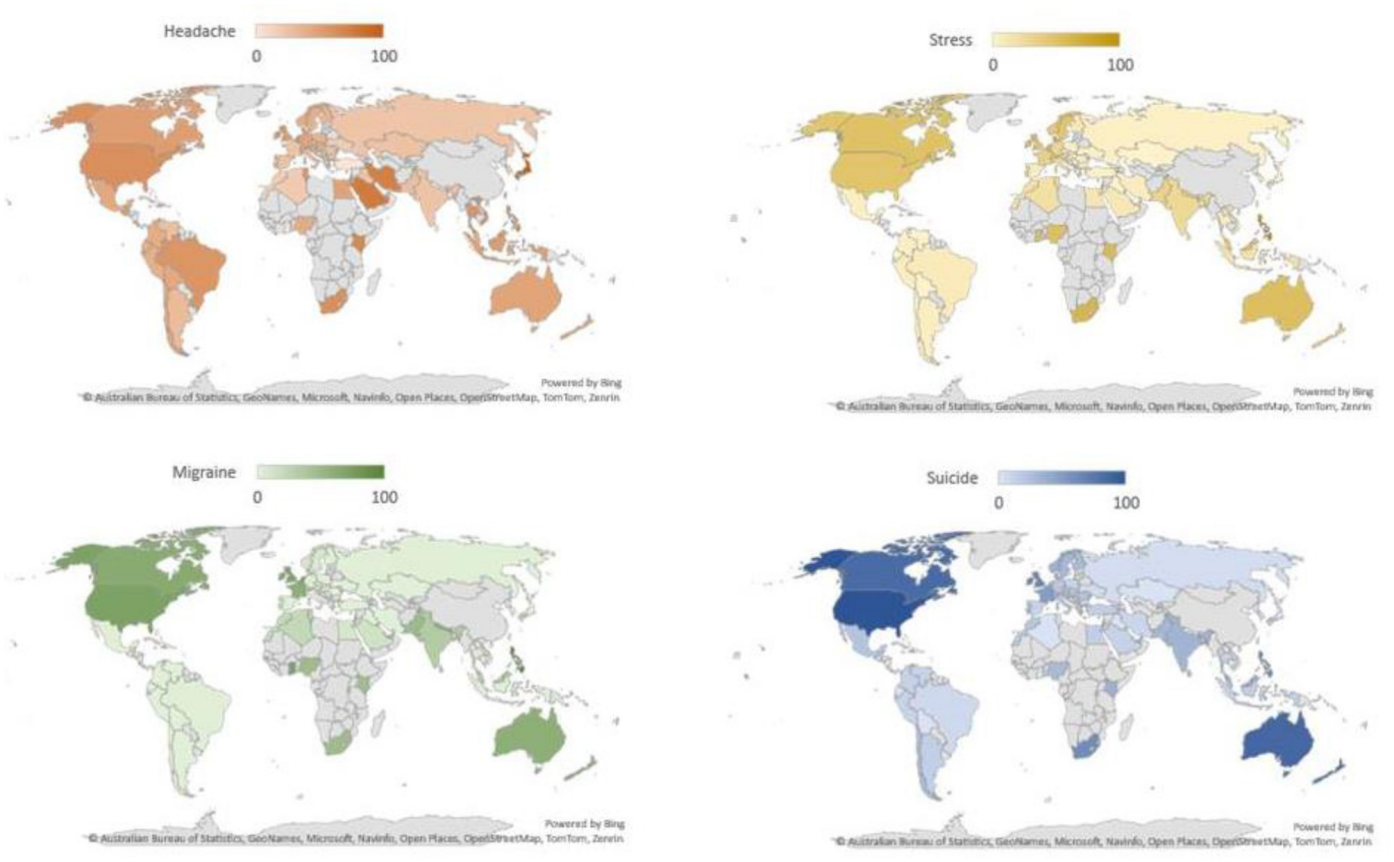
The individual key term searched worldwide headache, stress, migraine, and suicide.

This study indicates a significant association between search terms related to stress, headache, migraine, and suicide. The Pearson correlation coefficients and adjusted R-squared values demonstrated varying degrees of correlation between these terms (Akoglu, 2018; Humphreys et al., 2019; Rovetta, 2020). The strong correlation between “migraine” and “suicide” suggests a potential link between these two phenomena. It is worth noting that the linear regression model applied showed a good predictive value for search volume, especially in the case of stress, headache, and suicide. These findings support the hypothesis that the search trends for these terms are indeed correlated. Figures 2, 3 visually represent the relative search volume of these terms on Google Trends, offering insights into their seasonal and temporal variations. Over the study period from October 2013 to October 2023, a clear seasonal pattern emerged, with increased searches during the winter months and Geo map representation for all the terms illustrated in Figure 4. However, the study observed variations in the seasonal trends among different countries. Notably, some nations, such as Kenya, Philippines, and the United States, exhibited strong correlations between these search queries from month to month. Of particular concern is the rapid increase in suicide-related searches during certain time periods, notably in 2016–17 and during the COVID-19 pandemic period (2019–21). This suggests a potential link between stressful conditions, exacerbated by the pandemic, and an increase in searches related to suicide. It is noteworthy that the study found a higher prevalence of suicidal thoughts in the Indian population compared to the global average. The time series analysis revealed that certain countries, including the Philippines, experienced consistently high relative search volumes for all the terms over the study period. These findings could be indicative of the prevalence of stress-related conditions, headaches, migraines, and suicidal thoughts in these regions. During the COVID-19 pandemic, significant changes in search volumes were observed for stress, headache, and migraine, indicating the impact of the pandemic on individuals' mental health and wellbeing. While the search volume for “suicide” did not show a significant change, the study highlighted increased interest in these terms, particularly in countries like Kenya, Philippines, Jamaica, Trinidad and Tobago, the United States, and the Netherlands, suggesting that the pandemic had a profound impact on mental health awareness and concerns. In this study, the patient engaged in self-diagnosis, identifying themselves with conditions such as stress or headache or headache or suicide. This self-diagnosis could potentially introduce diagnostic errors, as it might blur the distinction between these conditions. The research, conducted using Google Trends data, reveals a noteworthy correlation between four terms that the public commonly searches for, namely “stress,” “headache,” “migraine,” and “suicide.” This suggests that patients have an understanding of the interconnection between these medical conditions (Wei et al., 2023). Consequently, when approaching these cases clinically, this relationship should be taken into account. It can serve as a valuable complement to the diagnostic process; preventing the overdiagnosis of such conditions and ensuring that primary headaches/migraine are not underdiagnosed, given the evident correlation between the search volumes, as indicated by this study. The current study is subject to certain limitations. Google Trends encompasses a vast database that includes searches conducted for a wide array of purposes, extending beyond health-related inquiries. Consequently, it is plausible that individuals may not be searching for these terms solely due to their personal experiences with these conditions. It emphasizes the practical implications of moderate correlations in public health surveillance, supporting our interpretation of the relationship between stress and suicide (R^2^ = 0.40) as meaningful despite being moderate (Johnson et al., 2011). Additionally, it should be noted that the search figures reported are relative values, not direct counts of search instances. Furthermore, the findings of this study are contingent on the internet literacy and, more specifically, health literacy of users, along with their access to the internet, potentially leading to a substantial bias in the representation of the population whose searches are included in this study. This underscores the reality that the volume of searches for a specific term offers no insight into the demographics of the population involved but rather provides only geographical data.

### 4.1 Stress as a precipitating factor for headaches and migraines

Our study highlights the established role of stress as a significant trigger for both headaches and migraines. The findings align with existing literature that supports the hypothesis that heightened stress levels are correlated with increased frequency and severity of these conditions. Numerous studies have demonstrated the neurobiological pathways linking stress to headache and migraine development, including the dysregulation of the hypothalamic-pituitary-adrenal (HPA) axis and the involvement of neurotransmitters such as serotonin and dopamine. Stress activates the HPA axis, leading to elevated levels of cortisol, which may contribute to neurovascular changes associated with headache and migraine pathophysiology. Moreover, chronic stress is known to induce sensitization of pain pathways, which can exacerbate the chronicity of headaches and migraines. This sensitization occurs due to neuroplastic changes within the central nervous system, ultimately leading to an increased perception of pain. Understanding these mechanisms reinforces the physiological basis of our findings and underscores the critical importance of stress management as a potential intervention. Implementing effective stress reduction strategies, such as cognitive-behavioral therapy, mindfulness practices, and lifestyle modifications, may help alleviate the burden of headaches and migraines among affected individuals.

### 4.2 Interconnectedness of headaches, migraines, and mental health

In our discussion, we also explore the comorbid relationship between migraines, chronic headaches, and mental health conditions such as anxiety and depression. Existing literature indicates a high prevalence of these comorbidities, which are often associated with an elevated risk of suicidal ideation and attempts. Our findings suggest that individuals suffering from migraines or chronic headaches may be particularly vulnerable to emotional distress, highlighting the need for a holistic approach to treatment that addresses both physical and mental health concerns. The bidirectional nature of these relationships further complicates the landscape. Studies have shown that individuals with a history of migraines are more likely to develop anxiety and depressive disorders. Conversely, those suffering from chronic stress may experience headaches or migraines as manifestations of their mental health struggles. This reciprocal relationship elucidates the heightened suicidal tendencies observed in our study population, emphasizing the critical need for integrated treatment approaches that consider both physical symptoms and underlying mental health issues.

### 4.3 Insights from infodemiology and public health implications

Utilizing Google Trends data, our study offers a novel perspective on public interest and concern regarding the interplay of stress, headaches, migraines, and suicide. This innovative approach captures real-time fluctuations in public discourse, serving as an early warning system for healthcare providers and policymakers. By identifying emerging trends in mental health and migraine-related issues, we can respond proactively to public health needs. Furthermore, our findings contextualize the potential for infodemiology to inform targeted public health campaigns aimed at raising awareness about the impacts of stress and the importance of mental health resources. Understanding public search behavior can enhance mental health interventions and support systems, ultimately leading to improved outcomes for individuals grappling with stress-related headaches and migraines.

### 4.4 Public health interventions

Our findings reveal a significant correlation between stress, headaches, migraines, and suicide, emphasizing the urgent need for public health campaigns that focus on stress management. By promoting awareness of the mental health implications associated with headaches and migraines, these campaigns can encourage individuals to seek help early and adopt preventive measures. Additionally, the strong association between chronic headaches, migraines, and mental health disorders highlights the necessity of integrated care approaches within healthcare settings. Training healthcare providers to recognize and address mental health concerns in patients with headache or migraine complaints could lead to more comprehensive treatment plans that address both physical and psychological health. Furthermore, our findings advocate for increased funding and resources dedicated to mental health services, providing policymakers with the evidence needed to justify improved access to care for populations burdened by migraines and headaches. The insights gained from this study can also guide the development of targeted interventions, such as community-based programs focused on stress reduction techniques, mindfulness training, cognitive-behavioral therapy, and support groups that emphasize effective coping strategies. Ultimately, we stress the importance of using data-driven research to inform policies that tackle the growing public health issues related to stress and its associated outcomes, allowing policymakers to formulate strategies that prioritize mental health awareness and interventions to reduce the prevalence of headaches and migraines.

### 4.5 Integrating mental health care into migraine management: addressing suicidality and comorbidities

The observed strong correlation between migraine and suicide highlights a critical need to integrate mental health care into migraine management. Current clinical guidelines emphasize the importance of evaluating and managing comorbid mental health conditions in patients with migraines. For example, the American Headache Society (AHS) Guidelines and the European Federation of Neurological Societies (EFNS) Guidelines both recommend comprehensive assessments of psychiatric comorbidities, including anxiety and depression, which are strongly associated with migraines and suicidal ideation. Similarly, the National Institute for Health and Care Excellence (NICE) Guidelines on Headaches in Over 12s: Diagnosis and Management highlight the necessity of identifying coexisting mental health disorders to provide holistic care. The Canadian Headache Society (CHS) Guidelines further advocate for integrating routine mental health assessments into clinical practice for migraine management. These recommendations align with the International Headache Society (IHS) Guidelines, which underscore the need for a multidisciplinary approach to address the complex interplay between migraines and mental health. Incorporating these guidelines into routine care can help identify individuals at higher risk of suicide and facilitate timely interventions, ultimately improving patient outcomes. These clinical guidelines for migraine often emphasize the evaluation of comorbid conditions such as anxiety and depression. These mental health conditions are well-established risk factors for suicidal ideation and attempts, necessitating a multidisciplinary approach. Incorporating routine psychological assessments in patients with migraines could help identify individuals at higher risk of suicide and facilitate timely interventions. Moreover, addressing chronic pain and its impact on mental health can alleviate the overall burden of these conditions.

### 4.6 Regional variations and underlying local factors

This study identifies notable regional variations in the Relative Search Volumes for stress, headaches, migraines, and suicide, with particularly high RSVs observed in the Philippines, India, and the United States. These differences underscore the need to consider local sociocultural and systemic factors in interpreting search behaviors. In the Philippines, the high RSVs for all terms may reflect increased public discourse around mental health, supported by growing awareness campaigns and evolving cultural attitudes toward discussing psychological wellbeing. Additionally, societal pressures and limited access to mental health services may drive individuals to seek online information. In India, the high RSVs for stress and suicide could be attributed to societal stigma surrounding mental health, which often prevents individuals from seeking formal medical care. Instead, many rely on the internet for anonymous self-education. Furthermore, structural barriers, such as unequal access to healthcare services in rural vs. urban areas, exacerbate this trend. In the United States, the high RSVs likely result from widespread internet access, a proactive culture of health literacy, and the availability of telehealth services. Online resources, including digital mental health tools, enable individuals to research their symptoms and seek support more readily. Healthcare accessibility also plays a critical role across these regions. Limited availability of mental health professionals in low-resource settings often leads to higher dependence on internet-based resources. Conversely, in nations with robust digital health infrastructures, such as the United States, higher RSVs reflect a population accustomed to leveraging technology for health-related concerns. These regional insights illustrate the intersection of cultural attitudes, healthcare infrastructure, and internet literacy in shaping search trends. Future research should aim to integrate qualitative assessments of these factors to better interpret the disparities observed in search behaviors and develop targeted interventions.

### 4.7 Pandemic-specific factors influencing RSV trends

The COVID-19 pandemic had a profound impact on public health, with significant increases in RSVs for stress, headaches, migraines, and suicide observed during this period. These trends can be attributed to several pandemic-specific factors:

*Lockdowns and social isolation*: Extended lockdowns and enforced social distancing measures disrupted daily routines and exacerbated feelings of loneliness and isolation. This isolation likely contributed to increased stress levels, as reflected in the surge of RSVs for terms like “stress” and “suicide.” For example, individuals experiencing mental health challenges during lockdowns may have turned to the internet for coping strategies and support.

*Economic uncertainty*: The pandemic triggered widespread economic instability, including job losses, financial insecurity, and reduced access to essential services. These stressors may explain the heightened RSVs for “stress” and “headache,” particularly in regions heavily affected by economic downturns, such as India and the Philippines.

*Healthcare access barriers*: Limited access to healthcare services due to overwhelmed systems and fear of infection may have driven individuals to seek online information about stress management, headache relief, and mental health support. This factor is especially relevant in countries where digital health tools were heavily utilized during the pandemic, such as the United States.

*Increased awareness through mental health campaigns*: Many governments and organizations launched mental health awareness campaigns during the pandemic to address rising mental health concerns. These campaigns, coupled with widespread media coverage, likely contributed to the increased RSVs for “stress” and “suicide,” as individuals sought information and resources online.

*Stress-Induced health conditions*: The pandemic's psychological toll likely contributed to physical symptoms, such as stress-induced headaches and migraines. This connection is reflected in the increased RSVs for “headache” and “migraine,” particularly during periods of peak pandemic-related restrictions.

*Digital behavior during lockdowns*: With more time spent online during lockdowns, individuals were more likely to use search engines to seek health-related information. This behavioral shift amplified search trends for stress, headaches, and suicide, as individuals increasingly relied on the internet for guidance and self-diagnosis.

### 4.8 Seasonal variations and their potential drivers

In regions such as the Philippines, the United States, and Europe, the end-of-year holiday season coincides with increased RSVs for stress and suicide. This may reflect heightened emotional distress due to financial pressures, social expectations, or feelings of loneliness during festive periods. Similarly, significant cultural events or festivals, such as Diwali in India, may contribute to spikes in stress-related searches as individuals manage celebratory demands and financial strain. Seasonal variations in weather conditions may also play a role in shaping search behaviors. For instance, colder winter months in countries like Canada, the United States, and Northern Europe often see higher RSVs for suicide and stress, potentially linked to seasonal affective disorder (SAD) and reduced daylight exposure. Conversely, warmer months in tropical regions like the Philippines and South Africa may exacerbate physical discomfort, contributing to increased RSVs for headaches and migraines. Academic and fiscal year deadlines may contribute to stress peaks in regions with structured education systems, such as the United Kingdom, Singapore, and India. Exams, performance reviews, and end-of-quarter work pressures often align with spikes in RSVs for stress and headache during specific months. Countries with prominent cultural or religious practices, such as Ramadan in Muslim-majority nations or Lent in Christian-majority countries, may experience shifts in RSVs due to fasting-related headaches, increased introspection, or heightened emotional states during these periods. During the COVID-19 pandemic, RSVs for stress, headaches, and suicide surged across most regions, reflecting the psychological and physical toll of lockdowns and economic instability. This underscores the role of external stressors in influencing search trends beyond regular seasonal patterns.

### 4.9 Current innovations

Our study introduces several key innovations that enhance understanding of the relationships among stress, headaches, migraines, and suicide. Primarily, this work utilizes Google Trends as an infodemiological tool, allowing us to analyze real-time fluctuations in public interest and concerns related to these interconnected health issues. This approach is innovative as it leverages publicly available search data to provide insights into collective behavior and concerns, which can offer a unique early indicator of mental health trends at the population level. Unlike traditional epidemiological studies, which often rely on retrospective data collection and clinical reports, our method captures immediate changes in interest or distress that may not yet be visible in healthcare settings. This real-time insight is particularly valuable for understanding the acute impact of external factors, such as economic downturns, public health crises, or societal stressors, on mental health-related searches. Additionally, by focusing on the relationships between these variables, our study bridges gaps between individual research areas, such as stress-related headaches, migraines, and suicidality, demonstrating their potential interconnectedness in public interest. This integrated analysis provides a new perspective on how these topics co-occur in the public mind, possibly indicating how societal stressors may intensify awareness or experience of both physical and psychological symptoms. Our methodological approach, including the use of relative search volume (RSV) to standardize data and track longitudinal patterns, adds another layer of innovation. It ensures that data is comparable across time periods, helping us discern trends rather than isolated spikes, which allows for a clearer understanding of persistent public interest or concern. This use of RSV also opens doors for future applications in public health, where similar analyses could help to monitor other emerging health concerns and guide timely interventions. These innovations collectively underscore the utility of digital surveillance tools in capturing and responding to health trends, especially in areas as complex and multifaceted as mental health and neurological disorders. By focusing on these immediate contributions, our study lays a foundation for future work that might build on these insights to inform public health responses and preventive measures in real time.

### 4.10 Multivariate analysis

The findings from the multivariate analysis reveal significant associations between stress, headache, and migraine RSVs with suicide RSV. However, the observed negative relationships are unexpected, given the anticipated positive correlations between stress-related conditions and suicide. This discrepancy could stem from several factors. The negative coefficients may reflect indirect relationships influenced by unmeasured confounders, such as increased public health awareness or interventions during periods of high RSVs for stress-related terms, which might reduce suicide-related searches. Additionally, the high R^2^ value and similar coefficient magnitudes suggest potential multicollinearity among predictors, where stress, headache, and migraine RSVs may overlap, distorting individual contributions. Variations in relationships across regions or time periods, such as during public health events like COVID-19, may also play a role, as stress-related RSVs might rise without a corresponding increase in suicide RSVs due to changes in societal behavior or reporting practices. Moreover, RSV data reflect search behavior rather than actual incidences, where individuals may seek information on managing stress or headaches rather than suicide-related topics. Finally, heightened public health campaigns addressing mental health could redirect individuals from suicidal ideation toward seeking stress-relief solutions, leading to apparent inverse correlations. These complexities highlight the need for further investigation to disentangle these dynamics.

### 4.11 Mechanisms linking migraines and suicide

**Figure 5:**
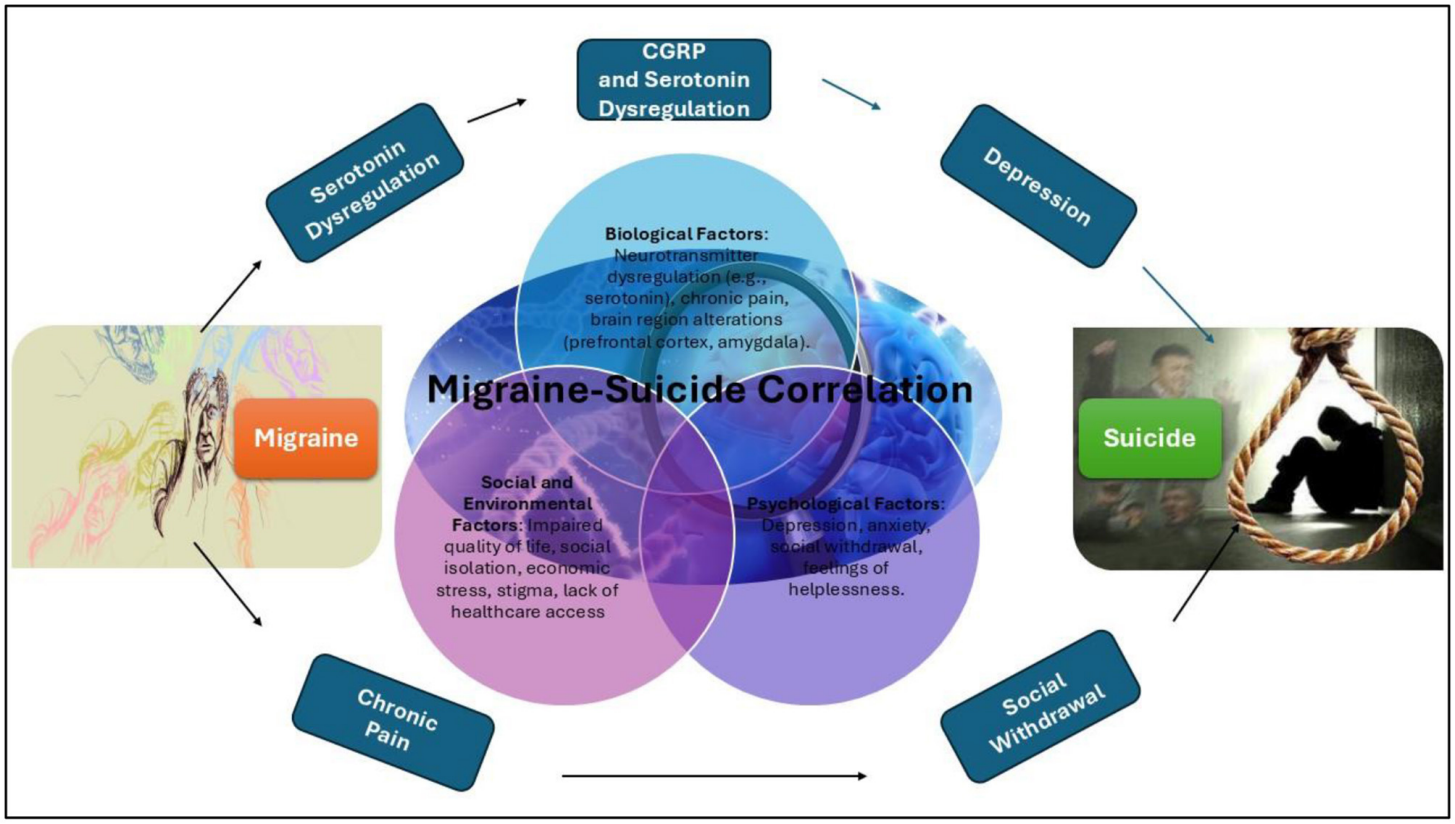
Mechanisms Linking Migraine and Suicide. The figure highlights the multifactorial mechanisms linking migraines to suicide, focusing on three key domains: biological factors, psychological factors, and social factors. Arrows illustrate the flow from migraines through these domains to suicide, emphasizing the interplay between neurological, psychological, and social influences on mental health outcomes.

The strong correlation was observed between migraines and suicide, biologically, migraines are linked to dysregulation of serotonin and other neurotransmitters, which are also implicated in depression and suicidality. Chronic pain from migraines can further alter brain regions involved in mood regulation, such as the prefrontal cortex and amygdala, heightening the risk of suicide. Psychologically, the frequent comorbidity of migraines with depression and anxiety a known risk factor for suicide combined with the debilitating and recurring nature of migraines, can exacerbate feelings of helplessness, frustration, and social withdrawal. Socially, chronic migraines often impair quality of life, leading to challenges in employment, relationships, and social roles, while economic stress and social isolation may drive suicidal ideation, particularly in regions with limited access to healthcare or social support systems illustrated in Figure 5. Additionally, stigma surrounding both mental health and neurological disorders may delay or prevent individuals from seeking timely intervention, compounding the psychological burden. While this study highlights the correlation, further research is needed to validate these mechanisms through clinical data and identify effective interventions.

## 5 Significance

This study provides a critical exploration into the interconnected dynamics among stress, headaches, migraines, and suicide by leveraging Google Trends data, underscoring the potential of infodemiology as a novel tool for mental health surveillance. By identifying patterns and temporal correlations in public interest related to these health issues, the research offers actionable insights for early intervention and crisis prevention. Importantly, these findings may enable public health agencies and policymakers to develop responsive, data-driven strategies aimed at mitigating stress-related health burdens. Furthermore, the study lays a foundation for using real-time search data to anticipate periods of heightened mental health risks, thereby supporting resource allocation and public awareness campaigns. This approach could ultimately contribute to more timely and targeted mental health interventions. This elaboration emphasizes the importance of the study in both advancing infodemiology and providing a practical foundation for addressing mental health challenges. Let me know if you'd like further refinement.

## 6 Limitation

- Google Trends data represents internet users and may not be representative of the broader population. It excludes individuals who don't use the internet or search engines, potentially introducing selection bias.
- Google Trends provides search volume data but doesn't reveal the specific context or reasons behind these searches.
- Google Trends data is based on a sample but doesn't explore the underlying causes of these fluctuations.
- The study suggests a strong correlation between migraines and suicide but does not delve deeper into the potential causes or factors contributing to this relationship.

*Representation bias*: Google Trends data inherently represents internet users, which may not accurately reflect the broader population. This limitation introduces a selection bias by excluding individuals who don't use the internet or search engines, potentially skewing the findings toward a digitally-active demographic. This limitation could lead to an overemphasis on trends among certain age groups, regions, or socioeconomic backgrounds that are more likely to use these platforms regularly.

*Lack of contextual insight*: Google Trends provides search volume data but lacks information on the specific motivations or contexts behind each search. This limitation restricts our understanding of why individuals are searching for terms related to stress, headaches, migraines, or suicide. Without these contextual details, interpreting search trends can be challenging, as we cannot definitively distinguish between information-seeking behavior, personal health concerns, or academic curiosity.

*Unexplored causes of seasonal patterns*: While the study identifies seasonal fluctuations in search volumes for certain terms, it does not explore the underlying causes of these patterns. This limitation leaves gaps in understanding the environmental, social, or psychological factors that may contribute to these seasonal trends. A more thorough examination of these drivers could enhance the study's ability to inform timely interventions.

*Correlation without causal analysis*: The observed correlation between migraines and suicide is a significant finding, yet this study does not delve into the potential causes or factors contributing to this relationship. Without examining underlying causal factors—such as mental health status, socioeconomic stressors, or medical comorbidities—the association remains observational. Understanding these contributing elements could be key to developing targeted prevention strategies.

The findings may be biased by disparities in internet access, reflecting populations with connectivity while excluding underserved or rural areas. Although RSVs standardize data across regions, they do not fully address digital divides. Future studies should incorporate offline data sources, such as surveys or clinical records, to enhance the understanding of global health trends. Google Trends RSV data reflects relative interest, not absolute search volumes or motivations behind search behavior. RSVs may be influenced by overall search activity and internet access variability. Thus, findings should be interpreted as trends in relative interest. Future studies could use qualitative methods, such as surveys or interviews, to explore factors driving search behavior and add context to these trends. Additionally, this study focuses on correlations between search terms (stress, headache, migraine, and suicide) using Google Trends data, providing insights into potential relationships but not causality. The observational nature of the data and reliance on RSVs limit the ability to infer causal pathways, as external factors like temporal trends or confounders may influence the results, requiring cautious interpretation.

## 7 Future perspectives

The research uncovers relationships between these search terms, potentially signifying hidden links among stress, headaches, migraines, and suicide. To understand affected populations, future studies may incorporate age, gender, and geography. Stress, migraines, and suicides present. Search traffic surged during COVID-19, showing external factors affect mental health. This data helps scientists and medics create crisis mental health treatments. This study links health issues, promoting public health. Understanding the links between stress, headaches, migraines, and suicide decreases stigma and promotes early treatment. Google Trends hospital admissions, surveys, and clinical diagnoses support it. Clinical data and online search trends explain health issues. Telemedicine and digital mental health services can aid online health information seekers as internet use rises. Project links pros to web searches. Study demonstrates mental health literacy is vital. Stress, migraines, and suicide are priorities. The research acknowledges national variances but emphasizes global trends. Geography impacts population plans. More online self-diagnosis and research raises privacy and data ethics concerns. Future research should address these issues and advise on health data safety. Health and information-seeking tendencies can be explained by longitudinal search research. Interdisciplinary teamwork, real-world health data, public health therapies, and awareness campaigns are needed for mental health. In the future, incorporating additional terms, such as 'depression,' 'anxiety,' and 'self-harm,' to provide a broader perspective on mental health-related search behaviors. This expanded approach would enable a more comprehensive analysis of the interplay between physical and mental health conditions, uncovering nuanced patterns and correlations across a wider range of factors. Future research should aim to bridge the digital divide by incorporating data from populations with limited internet access. This could involve the integration of offline health data, such as national surveys or healthcare utilization records, to validate and extend findings derived from online search trends. Exploring methods to adjust for regional disparities in digital literacy and access would further enhance the robustness and inclusivity of such studies.

## 8 Conclusion

The findings from the multivariate analysis reveal significant associations between stress, headache, and migraine RSVs with suicide RSV. The regression model demonstrated high explanatory power (R^2^ = 0.997), indicating that 99.7% of the variance in suicide RSV is explained by the independent variables. However, the observed negative relationships are unexpected, given the anticipated positive correlations between stress-related conditions and suicide. This discrepancy could stem from several factors. The negative coefficients may reflect indirect relationships influenced by unmeasured confounders, such as increased public health awareness or interventions during periods of high RSVs for stress-related terms, which might reduce suicide-related searches. Additionally, the high R^2^ value and similar coefficient magnitudes suggest potential multicollinearity among predictors, where stress, headache, and migraine RSVs may overlap, distorting individual contributions. Variations in relationships across regions or time periods, such as during public health events like COVID-19, may also play a role, as stress-related RSVs might rise without a corresponding increase in suicide RSVs due to changes in societal behavior or reporting practices. Moreover, RSV data reflect search behavior rather than actual incidences, where individuals may seek information on managing stress or headaches rather than suicide-related topics. Finally, heightened public health campaigns addressing mental health could redirect individuals from suicidal ideation toward seeking stress-relief solutions, leading to apparent inverse correlations. These findings underscore the importance of mental health awareness and the need for strategies to address the potential increase in stress-related conditions and suicidal thoughts during challenging times. Further research and interventions may be necessary to better understand and mitigate the impact of such stressors on public health.

## Data Availability

The raw data supporting the conclusions of this article will be made available by the authors, without undue reservation.
